# New Anti-Inflammatory Aporphine and Lignan Derivatives from the Root Wood of *Hernandia nymphaeifolia*

**DOI:** 10.3390/molecules23092286

**Published:** 2018-09-07

**Authors:** Chuan-Yen Wei, Shih-Wei Wang, Jin-Wang Ye, Tsong-Long Hwang, Ming-Jen Cheng, Ping-Jyun Sung, Tsung-Hsien Chang, Jih-Jung Chen

**Affiliations:** 1 Department of General Surgery, Taitung MacKay Memorial Hospital, Taitung City 950, Taiwan; lpshop@gmail.com; 2 Department of Medicine, Mackay Medical College, New Taipei City 252, Taiwan; shihwei@mmc.edu.tw; 3 Graduate Institute of Pharmaceutical Technology, Tajen University, Pingtung 907, Taiwan; jjc8506674@gmail.com; 4 Graduate Institute of Natural Products, School of Traditional Chinese Medicine, College of Medicine, Chang Gung University, Taoyuan 333, Taiwan; htl@mail.cgu.edu.tw; 5 Research Center for Chinese Herbal Medicine, Research Center for Food and Cosmetic Safety, Graduate Institute of Health Industry Technology, College of Human Ecology, Chang Gung University of Science and Technology, Taoyuan 333, Taiwan; 6 Department of Anesthesiology, Chang Gung Memorial Hospital, Taoyuan 333, Taiwan; 7 Bioresource Collection and Research Center (BCRC), Food Industry Research and Development Institute (FIRDI), Hsinchu 300, Taiwan; cmj@firdi.org.tw; 8 National Museum of Marine Biology and Aquarium, Pingtung 944, Taiwan; pjsung@nmmba.gov.tw; 9 Department of Medical Education and Research, Kaohsiung Veterans General Hospital, Kaohsiung 813, Taiwan; changth@vghks.gov.tw; 10 Faculty of Pharmacy, School of Pharmaceutical Sciences, National Yang-Ming University, Taipei 112, Taiwan; 11 Department of Medical Research, China Medical University Hospital, China Medical University, Taichung 404, Taiwan

**Keywords:** *Hernanadia nymphaeifolia*, Hernandiaceae, root wood, structure elucidation, aporphine, lignan, anti-inflammatory activity

## Abstract

A new aporphine, 3-hydroxyhernandonine (**1**) and a new lignin, 4′-*O*-demethyl-7-*O*-methyldehydropodophyllotoxin (**2**), have been isolated from the root wood of *Hernanadia nymphaeifolia*, together with thirteen known compounds (**3**–**15**). The structures of these compounds were determined through mass spectrometry (MS) and spectroscopic analyses. The known isolate, 2-*O*-methyl-7-oxolaetine (**3**), was first isolated from natural sources. Among the isolated compounds, 3-hydroxyhernandonine (**1**), 4′-*O*-demethyl-7-*O*-methyldehydropodophyllotoxin (**2**), hernandonine (**4**), oxohernangerine (**5**), and oxohernagine (**6**) displayed inhibition (IC_50_ values ≤5.72 μg/mL) of superoxide anion production by human neutrophils in response to formyl-l-methionyl-l-leucyl-l-phenylalanine/cytochalasin B (fMLP/CB). In addition, 3-hydroxyhernandonine (**1**), 4′-*O*-demethyl-7-*O*-methyldehydropodophyllotoxin (**2**), oxohernangerine (**5**), and oxohernagine (**6**) suppressed fMLP/CB-induced elastase release with IC_50_ values ≤5.40 μg/mL.

## 1. Introduction

*Hernanadia nymphaeifolia* (Presl) Kubitzki (Hernandiaceae) is an evergreen tree that is distributed in the tropical island shores of the Indian and western Pacific Oceans [1]. Its seed is used as a cathartic [2]. Various aporphines [3,4,5,6,7], isoquinolones [4,5], lignans [4,7,8], benzylisoquinoline [5], steroids [7], and their derivatives were isolated from this species in past studies. Many of these isolates display cytotoxic [4,5,8], vasorelaxing [6], antioxidant [6], and antiplatelet aggregation [7] activities.

**Figure 1:**
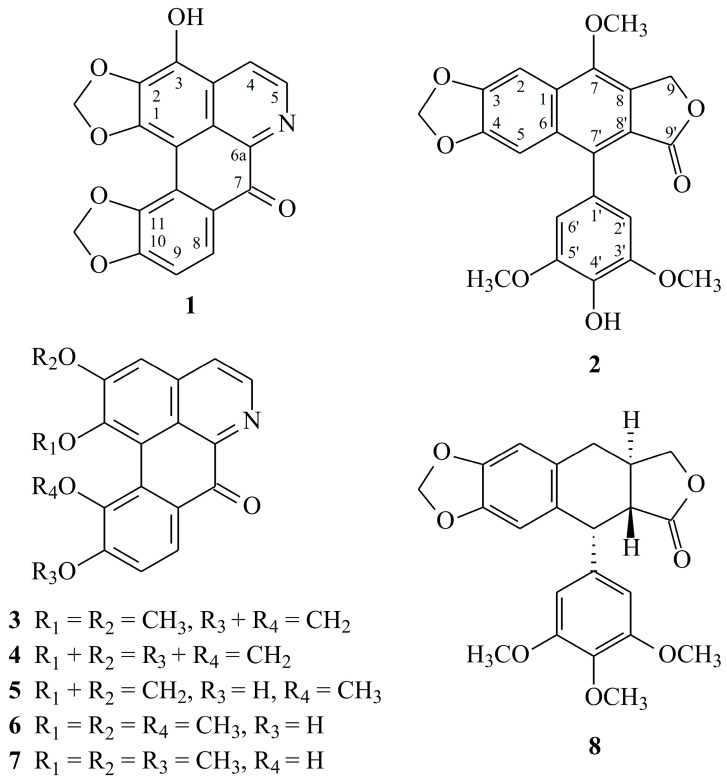

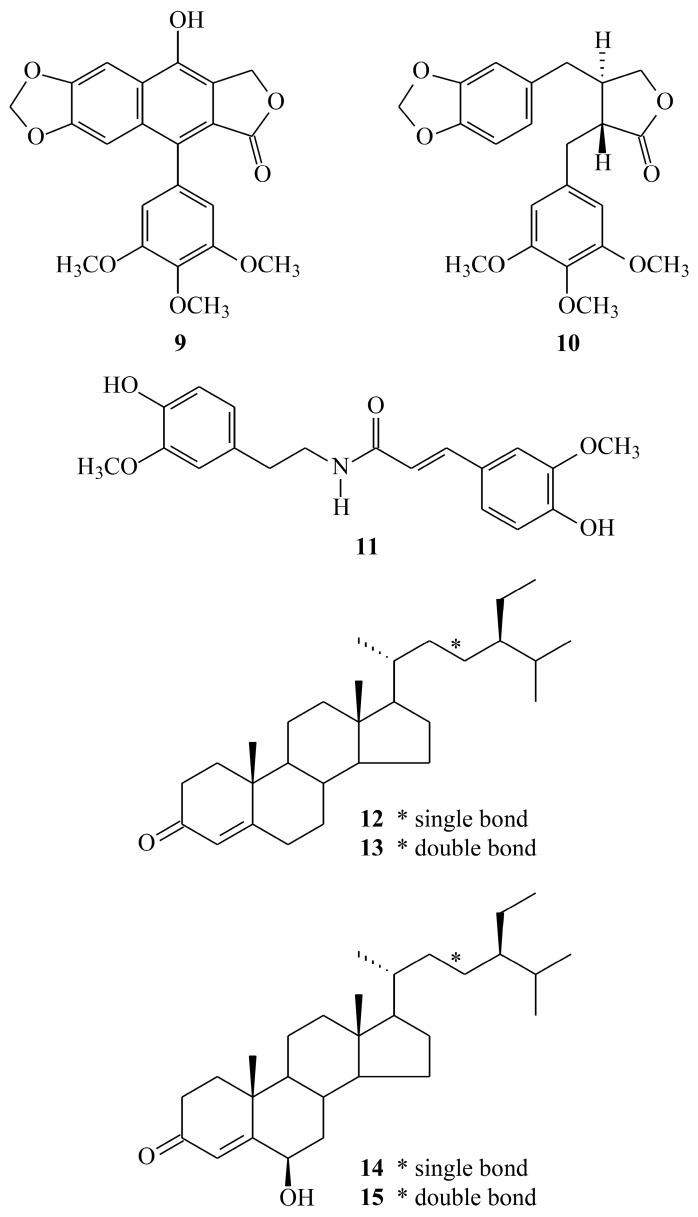
The chemical structures of compounds **1**–**15** isolated from *H. nymphaeifolia*.

The extensive or inappropriate activation of neutrophils leads to many inflammatory disorders such as chronic obstructive pulmonary disease (COPD), ischemia-reperfusion injury, asthma, rheumatoid arthritis, and metabolic diseases [9,10]. In response to various stimuli, activated neutrophils secrete a series of cytotoxins, such as granule proteases, bioactive lipids, and superoxide anion (O_2_^•–^), a precursor of other reactive oxygen species (ROS) [10,11,12]. The inhibition of the abnormal activation of neutrophils by medicines has been recommended as a way to improve inflammatory diseases. In our researches on the anti-inflammatory constituents of Formosan plants, numerous species have been screened for anti-inflammatory activity, and *H. nymphaeifolia* has been found to be an active species. A new aporphine, 3-hydroxyhernandonine (**1**), a new lignin, 4′-*O*-demethyl-7-*O*-methyldehydropodophyllotoxin (**2**), and thirteen known compounds (**3**–**15**) have been isolated and determined from the root wood of *Hernanadia nymphaeifolia,* and their structures are described in Figure 1.

This article describes the structural elucidation of new compounds **1** and **2** and the inhibitory effects of all isolates on elastase release and superoxide generation by human neutrophils.

## 2. Results and Discussion

### 2.1. Isolation and Structural Elucidation

Chromatographic purification of the CH_2_Cl_2_-soluble fraction of a MeOH extract of root wood of *H. nymphaeifolia* through a silica gel column, medium pressure liquid chromatography (MPLC), and preparative thin-layer chromatography (TLC) yielded two new (**1** and **2**) and thirteen known compounds (**3**–**15**) (Figure 1).

**Figure 2:**
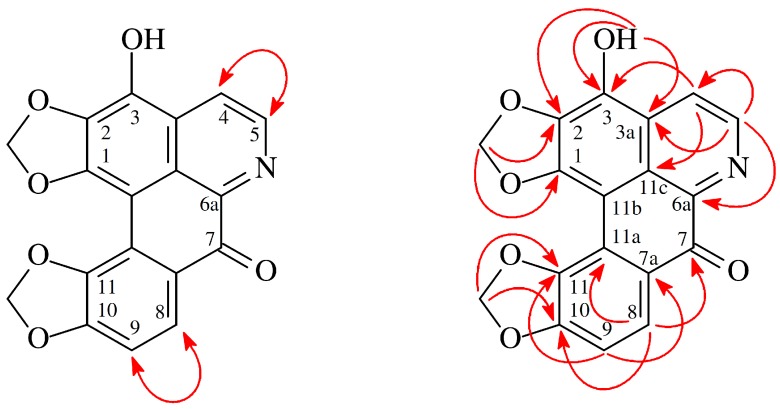
Key NOESY (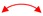) and HMBC (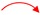) correlations of **1**.

The aporphine, 3-hydroxyhernandonine (**1**), was obtained as yellow needles. The electrospray ionization mass spectrometry (ESI-MS) (Figure S1) afford the quasi-molecular ion [M + Na]^+^ at *m*/*z* 358, implying a molecular formula of C_18_H_9_NO_6_Na, which was confirmed by the high-resolution (HR)-ESI-MS (*m*/*z* 358.0325 [M + Na]^+^, calcd 358.0328) (− 0.84 ppm) (Figure S2) and by the ^13^C-, ^1^H-, and distortionless enhancement by polarization transfer (DEPT) NMR data. IR absorptions for OH (3439 cm^−1^) and conjugated carbonyl (1646 cm^−1^) functions were observed. The ^1^H-NMR spectrum (Figure S3) of **1** showed the presence of a hydroxy group at δ_H_ 6.52 (1H, s, D_2_O exchangeable, OH-3), two methylenedioxy groups at δ_H_ 6.20 (2H, s, OCH_2_O-10,11) and 6.28 (2H, s, OCH_2_O-1,2), and two pairs of AB-doublets at δ_H_ 7.08 (1H, d, *J* = 8.5 Hz, H-9), 8.10 (1H, d, *J* = 5.0 Hz, H-4), 8.28 (1H, d, *J* = 8.5 Hz, H-8), and 8.88 (1H, d, *J* = 5.0 Hz, H-5). The ^1^H- and ^13^C-NMR (Figure S4) data of **1** were similar to those of hernandonine [13,14], except that the 3-hydroxy group [δ_H_ 6.52 (1H, s, D_2_O exchangeable)] of **1** replaced H-3 of hernandonine [13,14]. This was supported by HMBC correlations between OH-3 (δ_H_ 6.52) and C-2 (δ_C_ 139.2), as well as between C-3 (δ_C_ 148.4), and C-3a (δ_C_ 123.9). The full assignment of ^1^H- and ^13^C-NMR resonances was supported by DEPT, ^1^H–^1^H COSY (Figure S5), NOESY (Figure 2 and Figure S6), HMBC (Figure 2 and Figure S7), and HSQC (Figure S8) spectral analyses. Based on the above data, the structure of **1** was revealed as 3-hydroxyhernandonine. 

**Figure 3:**
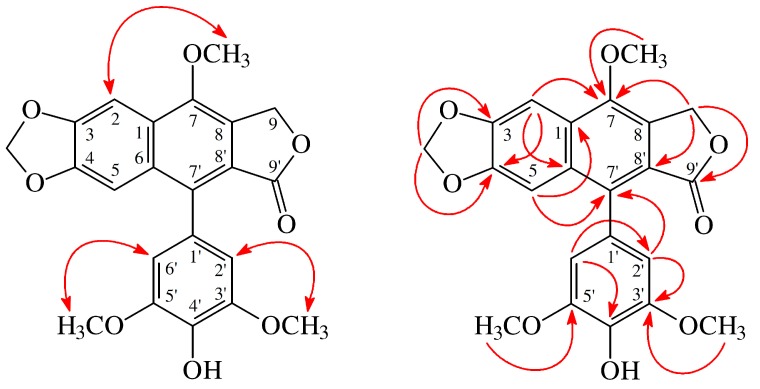
Key NOESY (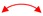) and HMBC (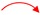) correlations of **2**.

4′-*O*-Demethyl-7-*O*-methyldehydropodophyllotoxin (**2**) was isolated as colorless needles. The ESI-MS (Figure S9) display the sodium adduct ion [M + Na]^+^ at *m*/*z* 433, hinting a molecular formula of C_22_H_18_O_8_, which was supported by the HR-ESI-MS (*m*/*z* 433.0898 [M + Na]^+^, calcd 433.0899) (– 0.23 ppm) (Figure S10). The IR spectrum showed the presence of OH (3452 cm^−1^) and γ-lactone carbonyl (1764 cm^−1^) groups. The ^1^H-NMR spectrum (Figure S11) of **2** showed the presence of three methoxy groups at δ_H_ 3.88 (6H, s, OMe-3′ and OMe-5′) and 4.09 (3H, s, OMe-7), a hydroxyl group at δ_H_ 5.65 (1H, br s, D_2_O exchangeable, OH-4′), a methylenedioxy group at δ_H_ 6.09 (2H, s, OCH_2_O), a γ-lactone methylene proton at 5.52 (2H, s, H-9), and four aromatic protons at δ_H_ 6.57 (2H, s, H-2′ and H-6′), 7.07 (1H, s, H-5), and 7.57 (1H, s, H-2). The ^1^H- and ^13^C-NMR (Figure S12) data of **2** were similar to those of 4′-*O*-demethyldehydropodophyllotoxin [15], except that the 7-methoxy groups [δ_H_ 4.09 (3H, s); δ_C_ 59.9 (OMe-7)] of **2** replaced the 7-OH group of 4′-*O*-demethyldehydropodophyllotoxin [15]. This was supported by NOESY correlations between OMe-7 (δ_H_ 4.09) and H-2 (δ_H_ 7.57) and by HMBC correlations between OMe-7 (δ_H_ 4.09) and C-7 (δ_C_ 148.5). According to the above evidence, the structure of **2** was elucidated as 4′-*O*-demethyl-7-*O*-methyldehydropodophyllotoxin. This was further affirmed by the ^1^H–^1^H-COSY (Figure S13), NOESY (Figure 3 and Figure S14), DEPT, HMBC (Figure 3 and Figure S15), and HSQC (Figure S16) experiments.

### 2.2. Structure Identification of the Known Isolates 

The known isolated compounds were readily confirmed by a comparison of spectroscopic and physical data (IR, UV, ^1^H-NMR, MS, and [α]_D_) with the literature values or corresponding authentic samples, and this included five aporphines, 2-*O*-methyl-7-oxolaetine (**3**) [16], hernandonine (**4**) [13,14], oxohernangerine (**5**) [17], oxohernagine (**6**) [17], and 7-oxonorisocorydine (**7**) [18], three lignans, (–)-deoxypodophyllotoxin (**8**) [19,20], dehydropodophyllotoxin (**9**) [20,21], (–)-yatein (**10**) [20], an amide, *N*-*trans*-feruloylmethoxytyramine (**11**) [22], four steroids, a mixture of β-sitostenone (**12**) [23] and stigmasta-4,22-dien-3-one (**13**) [23], and mixture of 6 β-hydroxystigmast-4-en-3-one (**14**) [24,25] and 6 β-hydroxystigmasta-4,22-dien-3-one (**15**) [24,25].

### 2.3. Biological Studies

Granule proteases (e.g., cathepsin G, elastase, and proteinase-3) and reactive oxygen species (ROS) (e.g., hydrogen peroxide and superoxide anion (O_2_^•−^)) generated by human neutrophils are involved in the pathogenesis of various NMR data [10,11,12,26]. The activities during neutrophil proinflammatory responses to isolates from the root wood of *H. nymphaeifolia* were assessed by inhibiting fMet-Leu-Phe/cytochalasin B (fMLP/CB)-induced O_2_^•–^ production and elastase release by human neutrophils. The inhibitory activity data on neutrophil proinflammatory responses are shown in Table 1. Diphenyleneiodonium and phenylmethylsulfonyl fluoride were employed as positive controls for O_2_^•–^ generation and elastase release, respectively [26]. From the results of our biological assays, the following conclusions can be summarized: (a) 3-hydroxyhernandonine (**1**), 4′-*O*-demethyl-7-*O*-methyldehydropodophyllotoxin (**2**), hernandonine (**4**), oxohernangerine (**5**), and oxohernagine (**6**) exhibited potent inhibition (IC_50_ ≤ 5.72 μg/mL) of superoxide anion (O_2_^•–^) generation by human neutrophils in response to fMLP/CB; (b) 3-hydroxyhernandonine (**1**), 4′-*O*-demethyl-7-*O*-methyldehydropodophyllotoxin (**2**), oxohernangerine (**5**), and oxohernagine (**6**) exhibited potent inhibition (IC_50_ ≤ 5.40 μg/mL) of fMLP-induced elastase release; (c) the aporphine alkaloid, 3-hydroxyhernandonine (**1**) (with a 3-hydroxy group), exhibited more effective inhibition than its analogue, hernandonine (**4**) (without any substitutant at C-3), against fMLP-induced O_2_^•–^ generation and elastase release; (d) oxohernagine (**6**) (with 10-hydroxy and 11-methoxy groups) exhibited more effective inhibition of fMLP-induced O_2_^•–^ generation and elastase release than its analogue, 7-oxonorisocorydine (**7**) (with 11-hydroxy and 10-methoxy groups; (e) the lignan compound, 4′-*O*-demethyl-7-*O*-methyldehydroodophyllotoxin (**2**) (with 7-methoxy and 4′-hydroxy groups) exhibited more effective inhibition of fMLP-induced O_2_^•–^ generation and elastase release than its analogue, dehydropodophyllotoxin (**9**) (with 7-hydroxy and 4′-methoxy groups); (f) oxohernangerine (**5**) was the most effective among these compounds, with an IC_50_ value of 2.65 ± 0.97 μg/mL, against fMLP-induced superoxide anion generation; (g) 3-hydroxyhernandonine (**1**) was the most effective among the isolates, with an IC_50_ value of 3.93 ± 0.48 μg/mL against fMLP-induced elastase release.

## 3. Experimental Section

### 3.1. General Procedures

Melting points were determined on a Yanaco micromelting point apparatus and were uncorrected. Ultraviolet (UV) spectra were measured on a Jasco UV-240 spectrophotometer. Optical rotations were acquired using a Jasco DIP-370 polarimeter (Japan Spectroscopic Corporation, Tokyo, Japan) in CHCl_3_. Infrared (IR) spectra (KBr or neat) were recorded on a Perkin Elmer 2000 FT-IR spectrometer (Perkin Elmer Corporation, Norwalk, CT, USA). Nuclear magnetic resonance (NMR) spectra, including correlation spectroscopy (COSY), nuclear overhauser effect spectrometry (NOESY), heteronuclear multiple-bond correlation (HMBC) experiments, and heteronuclear single-quantum coherence (HSQC), were obtained using a Varian Inova 500 spectrometer (Varian Inc., Palo Alto, CA, USA) operating at 500 MHz (^1^H) and 125 MHz (^13^C), respectively, with chemical shifts given in ppm (δ) and applying tetramethylsilane (TMS) as an internal standard. Electrospray ionization (ESI) and high-resolution electrospray ionization (HRESI)-mass spectra were recorded on a VG Platform Electrospray ESI/MS mass spectrometer (Fison, Villeurbanne, France) or a Bruker APEX II (Bruker, Bremen, Germany). Silica gel (70–230, 230–400 mesh, Merck) was used for column chromatography (CC). Silica gel 60 F-254 (Merck, Darmstadt, Germany) was employed for thin-layer chromatography (TLC) and preparative thin-layer chromatography (PTLC).

### 3.2. Plant Material

The root wood of *Hernanadia nymphaeifolia* (Presl) Kubitzki (Hernandiaceae) was collected from Mudan Township, Pingtung County, Taiwan, in August 2008 and identified by Prof. I.-S. Chen. A voucher specimen (Chen 5521) was deposited in the Herbarium of School of Pharmacy, Kaohsiung Medical University, Kaohsiung, Taiwan.

### 3.3. Extraction and Isolation

The dried root wood (5.1 kg) of *H. nymphaeifolia* was sliced and extracted three times with MeOH (40 L each) for three days. The extract was concentrated under reduced pressure at 35 °C, and the residue (386 g) was partitioned between CH_2_Cl_2_ and H_2_O (1:1) to provide the CH_2_Cl_2_-soluble fraction (fraction A; 87 g). Fraction A (87 g) was purified by CC (3.9 kg of SiO_2_, 70–230 mesh; CH_2_Cl_2_/MeOH gradient) to produce 12 fractions: A1–A12. Fraction A3 (7.5 g) was subjected to CC (340 g of SiO_2_, 230–400 mesh; CH_2_Cl_2_/acetone 30:1–0:1, 900 mL fractions) to give 11 subfractions: A3-1–A3-11. Fraction A3-4 (340 mg) was purified by MPLC (silica column, CH_2_Cl_2_/acetone 8:1–0:1) to produce eight subfractions (each 250 mL, A3-4-1–A3-4-8). Fraction A3-4-4 (46 mg) was purified by preparative TLC (silica gel, CHCl_3_/MeOH, 10:1) to obtain a mixture of β-sitostenone (**12**) and stigmasta-4,22-dien-3-one (**13**) (8.5 mg). Fraction A5 (6.9 g) was subjected to CC (365 g of SiO_2_, 230–400 mesh; CH_2_Cl_2_/MeOH 15:1–0:1, 950 mL fractions) to form ten subfractions: A5-1–A5-10. Fraction A5-3 (625 mg) was purified by CC (28 g of SiO_2_, 230–400 mesh, CHCl_3_/acetone (7:1–0:1), 250 mL fractions) to give nine subfractions: A5-3-1–A5-3-9. Fraction A5-3-5 (88 mg) was further purified by preparative TLC (SiO_2_; CH_2_Cl_2_/acetone 8:1) to yield a mixture of 6β-hydroxystigmast-4-en-3-one (**14**) and 6β-hydroxystigmasta-4,22-dien-3-one (**15**) (3.7 mg). Fraction A7 (7.3 g) was subjected to CC (330 g of SiO_2_, 230–400 mesh; CHCl_3_/MeOH 10:1–0:1, 800 mL fractions) to give nine subfractions: A7-1–A7-9. A part (142 mg) of fraction A7-2 was further purified by preparative TLC (SiO_2_; CH_2_Cl_2_/MeOH 15:1) to form (–)-deoxypodophyllotoxin (**8**) (7.2 mg). A part (133 mg) of fraction A7-3 was further purified by preparative TLC (SiO_2_; CHCl_3_/MeOH 12:1) to yield (–)-yatein (**10**) (5.1 mg). A part (136 mg) of fraction A7-4 was further purified by preparative TLC (SiO_2_; CHCl_3_/MeOH 9:1) to obtain 2-*O*-methyl-7-oxolaetine (**3**) (5.3 mg). A part (118 mg) of fraction A7-6 was further purified by preparative TLC (SiO_2_; CH_2_Cl_2_/MeOH 5:1) to produce 7-oxonorisocorydine (**7**) (6.5 mg). Fraction A7-7 (650 mg) was purified by MPLC (silica column, CH_2_Cl_2_/MeOH 9:1–0:1) to form seven subfractions (each 170 ml, A7-7-1–A7-7-7). A part (112 mg) of fraction A7-7-4 was further purified by preparative TLC (SiO_2_; CHCl_3_/acetone 5:1) to form 4′-*O*-demethyl-7-*O*-methyldehydropodophyllotoxin (**2**) (5.5 mg). A part (125 mg) of fraction A7-7-5 was purified by preparative TLC (SiO_2_; CH_2_Cl_2_/acetone 4:1) to obtain dehydropodophyllotoxin (**9**) (6.9 mg). A part (122 mg) of fraction A7-8 was purified by preparative TLC (SiO_2_; CH_2_Cl_2_/EtOAc 2:1) to yield *N*-*trans*-feruloylmethoxytyramine (**11**) (4.9 mg). Fraction A8 (7.2 g) was subjected to CC (325 g of SiO_2_, 230–400 mesh; CH_2_Cl_2_/MeOH 8:1–0:1, 850 mL fractions) to give ten subfractions: A8-1–A8-10. Fraction A8-2 (530 mg) was purified by MPLC (silica column, CHCl_3_/MeOH 7:1–0:1) to form six subfractions (each 180 ml, A8-2-1–A8-2-6). Fraction A8-2-4 (83 mg) was further purified by preparative TLC (SiO_2_; CH_2_Cl_2_/acetone 6:1) to obtain hernandonine (**4**) (8.2 mg). Fraction A8-5 (135 mg) was further purified by preparative TLC (SiO_2_; CH_2_Cl_2_/MeOH 4:1) to obtain oxohernagine (**6**) (7.1 mg). Fraction A8-6 (135 mg) was further purified by preparative TLC (SiO_2_; CHCl_3_/MeOH 3:1) to yield oxohernangerine (**5**) (6.5 mg). Fraction A9 (6.4 g) was subjected to CC (290 g of SiO_2_, 230–400 mesh; CHCl_3_/MeOH 6:1–0:1, 1 L fractions) to obtain eight subfractions: A9-1–A9-8. A part (142 mg) of fraction A9-3 was purified by preparative TLC (SiO_2_; CHCl_3_/MeOH 5:1) to obtain 3-hydroxyhernandonine (**1**) (4.5 mg). A part (105 mg) of fraction A9-5 was purified by preparative TLC (SiO_2_; CH_2_Cl_2_/MeOH 4:1) to obtain oxohernangerine (**5**) (5.9 mg).

*3-Hydroxyhernandonine* (**1**): yellow needles; m.p. 268–270 °C (MeOH); UV (MeOH): λ_max_ (log ε) = 220 (3.90), 268 (3.79), 284 (3.78), 343 (3.46), 362 (3.47) nm; IR (KBr): υ_max_ = 3315 (OH), 1652 (C=O), 1060, 969 (OCH_2_O) cm^−1^; ^1^H-NMR (CDCl_3_, 500 MHz): δ 6.20 (2H, s, OCH_2_O-10,11), 6.28 (2H, s, OCH_2_O-1,2), 6.52 (1H, s, D_2_O exchangeable, OH-3), 7.08 (1H, d, *J* = 8.5 Hz, H-9), 8.10 (1H, d, *J* = 5.0 Hz, H-4), 8.28 (1H, d, *J* = 8.5 Hz, H-8), 8.88 (1H, d, *J* = 5.0 Hz, H-5); ^13^C-NMR (CDCl_3_, 125 MHz): δ 101.3 (OCH_2_O-1,2), 101.7 (OCH_2_O-10,11), 108.6 (C-9), 114.9 (C-11b), 118.7 (C-8), 119.1 (C-4), 122.3 (C-11a), 122.9 (C-11c), 123.9 (C-3a), 127.6 (C-7a), 139.2 (C-2), 145.2 (C-5), 145.8 (C-11), 148.4 (C-3), 149.6 (C-1), 150.4 (C-10), 157.3 (C-6a), 182.5 (C-7); ESI-MS: *m*/*z* = 358 [M + Na]^+^; HR-ESI-MS: *m*/*z* = 358.0325 [M + Na]^+^ (calcd for C_18_H_9_NO_6_Na, 358.0328).

*4′-O-Demethyl-7-O-methyldehydropodophyllotoxin* (**2**): colorless needles; m.p. 273–275 °C (MeOH); UV (MeOH): λ_max_ (log ε) = 223 (4.47), 262 (4.58), 321 (3.98), 355 (3.69) nm; IR (KBr): υ_max_ = 3452 (OH), 1764 (C=O) cm^−1^; ^1^H-NMR (CDCl_3_, 500 MHz): δ 3.88 (6H, s, OMe-3′ and OMe-5′), 4.09 (3H, s, OMe-7), 5.52 (2H, s, H-9), 5.65 (1H, br s, D_2_O exchangeable, OH-4′), 6.09 (2H, s, OCH_2_O), 6.57 (2H, s, H-2′, and H-6′), 7.07 (1H, s, H-5), 7.57 (1H, s, H-2); ^13^C-NMR (CDCl_3_, 125 MHz): δ 56.0 (OMe-3′), 56.0 (OMe-5′), 59.9 (OMe-7), 66.4 (C-9), 98.4 (C-2), 101.8 (OCH_2_O), 103.9 (C-5), 107.7 (C-2′), 107.7 (C-6′), 119.5 (C-8′), 125.8 (C-8), 127.8 (C-6), 130.4 (C-1), 132.2 (C-1′), 133.6 (C-4′), 137.7 (C-7′), 148.5 (C-7), 148.9 (C-4), 148.9 (C-3′), 148.9 (C-5′), 150.0 (C-3), 169.3 (C-9′); ESI-MS: *m*/*z* = 433 [M + Na]^+^; HR-ESI-MS: *m*/*z* = 433.0898 [M + Na]^+^ (calcd for C_22_H_18_O_8_Na, 433.0899).

*2-O-Methyl-7-oxolaetine* (**3**): yellow needles; m.p. > 300 °C (MeOH); UV (MeOH): λ_max_ (log ε) = 221 (4.49), 266 (4.33), 362 (4.00), 427 (3.95) nm; IR (KBr): υ_max_ = 1652 (C=O) cm^−1^; ^1^H-NMR (CDCl_3_, 400 MHz): δ 3.93 (3H, s, OMe-1), 6.21 (2H, s, OCH_2_O-10,11), 7.08 (1H, d, *J* = 8.4 Hz, H-9), 7.21 (1H, s, H-3), 7.77 (1H, d, *J* = 5.2 Hz, H-4), 8.24 (1H, d, *J* = 8.4 Hz, H-8), 8.86 (1H, d, *J* = 5.2 Hz, H-5); ESI-MS: *m*/*z* = 358 [M + Na]^+^; HR-ESI-MS: *m*/*z* = 358.0692 [M + Na]^+^ (calcd for C_19_H_13_O_5_Na, 358.0691).

*Hernandonine* (**4**): yellow needles; m.p. > 350 °C (CH_2_Cl_2_-MeOH); UV (MeOH): λ_max_ (log ε) = 222 (4.50), 265 (4.34), 295 (sh, 3.90), 312 (sh, 3.63), 364 (4.02), 428 (3.97) nm; IR (KBr): υ_max_ = 1651 (C=O), 1062, 971 (OCH_2_O) cm^−1^; ^1^H-NMR (CDCl_3_, 500 MHz): δ 6.20 (2H, s, OCH_2_O-10,11), 6.28 (2H, s, OCH_2_O-1,2), 7.08 (1H, d, *J* = 8.5 Hz, H-9), 7.21 (1H, s, H-3), 7.74 (1H, d, *J* = 5.0 Hz, H-4), 8.29 (1H, d, *J* = 8.5 Hz, H-8), 8.85 (1H, d, *J* = 5.0 Hz, H-5); ESI-MS: *m*/*z* = 342 [M + Na]^+^.

*Oxohernangerine* (**5**): yellow prisms; m.p. 257–258 °C (MeOH); UV (MeOH): λ_max_ (log ε) = 211 (4.55), 252 (4.46), 268 (sh, 4.42), 316 (3.87), 362 (4.04), 408 (4.02), 477 (3.55) nm; IR (KBr): υ_max_ = 3415 (OH), 1642 (C=O) cm^−1^; ^1^H-NMR (CDCl_3_, 400 MHz): δ 3.68 (3H, s, OMe-11), 6.32 (2H, s, OCH_2_O-1,2), 7.24 (1H, d, *J* = 8.4 Hz, H-9), 7.27 (1H, s, H-3), 7.76 (1H, d, *J* = 5.2 Hz, H-4), 8.37 (1H, d, *J* = 8.4 Hz, H-8), 8.86 (1H, d, *J* = 5.2 Hz, H-5); ESI-MS: *m*/*z* = 344 [M + Na]^+^.

*Oxohernagine* (**6**): yellow prisms; m.p. 253–255 °C (MeOH); UV (MeOH): λ_max_ (log ε) = 213 (4.51), 274 (4.41), 361 (3.95), 403 (3.92) nm; IR (KBr): υ_max_ = 3424 (OH), 1650 (C=O) cm^−1^; ^1^H-NMR (CDCl_3_, 400 MHz): δ 3.54 (3H, s, OMe-1), 3.76 (3H, s, OMe-11), 4.11 (3H, s, OMe-2), 7.21 (1H, s, H-3), 7.22 (1H, d, *J* = 8.4 Hz, H-9), 7.77 (1H, d, *J* = 5.2 Hz, H-4), 8.31 (1H, d, *J* = 8.4 Hz, H-8), 8.86 (1H, d, *J* = 5.2 Hz, H-5); ESI-MS: *m*/*z* = 360 [M + Na]^+^.

*7-Oxonorisocorydine* (**7**): yellow needles; m.p. 250–252 °C (EtOAc); UV (MeOH): λ_max_ (log ε) = 212 (4.49), 273 (4.40), 362 (3.94), 403 (3.90) nm; IR (KBr): υ_max_ = 3385 (OH), 1653 (C=O) cm^−1^; ^1^H-NMR (CDCl_3_, 400 MHz): δ 3.53 (3H, s, OMe-1), 4.03 (3H, s, OMe-10), 4.08 (3H, s, OMe-2), 7.14 (1H, d, *J* = 8.4 Hz, H-9), 7.23 (1H, s, H-3), 7.77 (1H, d, *J* = 5.2 Hz, H-4), 8.28 (1H, d, *J* = 8.4 Hz, H-8), 8.86 (1H, d, *J* = 5.2 Hz, H-5); ESI-MS: *m*/*z* = 360 [M + Na]^+^.

*(–)-Deoxypodophyllotoxin* (**8**): colorless needles; m.p. 168–170 °C (MeOH); UV (MeOH): λ_max_ (log ε) = 212 (4.62), 291 (3.68) nm; IR (KBr): υ_max_ = 1765 (C=O), 1581, 1502, 1474 (aromatic ring C=C stretch), 1032, 941 (OCH_2_O) cm^−1^; ^1^H-NMR (CDCl_3_, 500 MHz): δ 2.73 (3H, m, H-7, H-8, and H-8′), 3.07 (1H, m, H-7), 3.75 (6H, s, OMe-3′, and OMe-5′), 3.80 (3H, s, OMe-4′), 3.92 (1H, m, H-9), 4.46 (1H, m, H-9), 4.60 (1H, d, *J* = 3.5 Hz, H-7′), 5.93, 5.95 (each 1H, each d, *J* = 1.5 Hz, OCH_2_O), 6.34 (2H, s, H-2′, and H-6′), 6.52 (1H, s, H-5), 6.67 (1H, s, H-2); ESI-MS: *m*/*z* = 421 [M + Na]^+^.

*Dehydropodophyllotoxin* (**9**): colorless needles; m.p. 264–266 °C (CH_2_Cl_2_-MeOH); UV (MeOH): λ_max_ (log ε) = 262 (4.57), 311 (3.95), 321 (3.97) nm; IR (KBr): υ_max_ = 3421 (OH), 1762 (C=O) cm^−1^; ^1^H-NMR (CDCl_3_, 500 MHz): δ 3.83 (6H, s, OMe-3′, and OMe-5′), 3.95 (3H, s, OMe-4′), 5.37 (2H, br s, H-9), 5.68 (1H, br s, D_2_O exchangeable, OH-7), 6.10 (2H, s, OCH_2_O), 6.52 (2H, s, H-2′, and H-6′), 7.09 (1H, s, H-5), 7.49 (1H, s, H-2); ESI-MS: *m*/*z* = 433 [M + Na]^+^.

*(–)-Yatein* (**10**): yellowish solid (MeOH); UV (MeOH): λ_max_ (log ε) = 212 (4.35), 230 (sh, 3.93), 287 (3.47) nm; IR (KBr): υ_max_ = 1764 (C=O), 1591, 1502, 1488 (aromatic ring C=C stretch), 1037, 925 (OCH_2_O) cm^−1^; ^1^H-NMR (CDCl_3_, 400 MHz): δ 2.49 (1H, m, H-8), 2.53 (1H, m, H-7α), 2.58 (1H, m, H-8′), 2.62 (1H, dd, *J* = 13.2, 6.4 Hz, H-7β), 2.89 (1H, dd, *J* = 14.0, 6.2 Hz, H-7′α), 2.93 (1H, dd, *J* = 14.0, 5.2 Hz, H-7′β), 3.82 (6H, s, OMe-3′, and OMe-5′), 3.83 (3H, s, OMe-4′), 3.88 (1H, dd, *J* = 9.2, 7.6 Hz, H-9β), 4.18 (1H, dd, *J* = 9.2, 7.2 Hz, H-9α), 5.93, 5.94 (each 1H, each d, *J* = 1.2 Hz, OCH_2_O), 6.36 (2H, s, H-2′, and H-6′), 6.46 (1H, d, *J* = 1.6 Hz, H-2), 6.47 (1H, dd, *J* = 7.6, 1.6 Hz, H-6), 6.69 (1H, d, *J* = 7.6 Hz, H-5); ESI-MS: *m*/*z* = 423 [M + Na]^+^.

*N-trans-Feruloylmethoxytyramine* (**11**): white needles; m.p. 112–114 °C (CHCl_3_-MeOH); UV (MeOH): λ_max_ (log ε) = 221 (3.61), 290 (2.86), 319 (3.34) nm; IR (KBr): υ_max_ = 3362 (OH), 1652 (C=O) cm^−1^; ^1^H-NMR (CDCl_3_, 400 MHz): δ 2.82 (2H, t, *J* = 6.8 Hz, H-11), 3.62 (2H, q, *J* = 6.8 Hz, H-10), 3.88 (3H, s, OMe-14), 3.92 (3H, s, OMe-3), 5.52 (1H, br t, *J* = 6.8 Hz, D_2_O exchangeable, NH), 5.53 (1H, s, D_2_O exchangeable, OH), 5.79 (1H, s, D_2_O exchangeable, OH), 6.16 (1H, d, *J* = 15.6 Hz, H-8), 6.71 (1H, dd, *J* = 8.0, 1.6 Hz, H-17), 6.73 (1H, d, *J* = 1.6 Hz, H-13), 6.87 (1H, d, *J* = 8. Hz, H-16), 6.90 (1H, d, *J* = 8.4 Hz, H-5), 6.97 (1H, d, *J* = 1.6 Hz, H-2), 7.04 (1H, dd, *J* = 8.4, 1.6 Hz, H-5), 7.53 (1H, d, *J* = 15.6 Hz, H-7); ESI-MS: *m*/*z* = 366 [M + Na]^+^.

*Mixture of β-Sitostenone* (**12**) and stigmasta-4,22-dien-3-one (**13**): colorless needles; m.p. 88–90 °C (MeOH); [α${]}_{D}^{25}$ = +85.8° (*c* 0.18, CHCl_3_); UV (MeOH): λ_max_ (log ε) = 242 (4.21); IR (KBr): υ_max_ = 1685 (C=O) cm^−1^; ^1^H-NMR (CDCl_3_, 400 MHz) of **12**: δ 0.70 (3H, s, H-18), 0.81 (3H, d, *J* = 6.8 Hz, H-27), 0.83 (3H, d, *J* = 6.8 Hz, H-26), 0.86 (3H, t, *J* = 7.2 Hz, H-29), 0.92 (3H, d, *J* = 6.4 Hz, H-21), 1.18 (3H, s, H-19), 5.71 (1H, s, H-4); ^1^H-NMR (CDCl_3_, 400 MHz) of **13**: δ 0.72 (3H, s, H-18), 0.79 (3H, d, *J* = 6.8 Hz, H-27), 0.82 (3H, t, *J* = 7.2 Hz, H-29), 0.83 (3H, d, *J* = 6.8 Hz, H-26), 1.02 (3H, d, *J* = 6.8 Hz, H-21), 1.18 (3H, s, H-19), 5.02 (1H, dd, *J* = 15.2, 8.8 Hz, H-23), 5.14 (1H, dd, *J* = 15.2, 8.8 Hz, H-22), 5.71 (1H, s, H-4); ESI-MS of **12**: *m*/*z* = 435 [M + Na]^+^; ESI-MS of **13**: *m*/*z* = 433 [M + Na]^+^. 

*Mixture of6β-Hydroxystigmast-4-en-3-one* (**14**) and 6β-hydroxystigmasta-4,22-dien-3-one (**15**): colorless needles; m.p. 208–209 °C (CH_2_Cl_2_-MeOH); [α${]}_{D}^{25}$ = + 29.7° (*c* 0.17, CHCl_3_); UV (MeOH): λ_max_ (log ε) = 235 (4.11) nm; IR (KBr): υ_max_ = 3412 (OH), 1679 (C=O) cm^−1^; ^1^H-NMR (CDCl_3_, 400 MHz) of **14**: δ 0.74 (3H, s, H-18), 0.81 (3H, d, *J* = 6.8 Hz, H-27), 0.84 (3H, d, *J* = 7.2 Hz, H-26), 0.87 (3H, t, *J* = 7.2 Hz, H-29), 0.92 (3H, d, *J* = 6.4 Hz, H-21), 1.38 (3H, s, H-19), 4.35 (1H, br s, H-6), 5.82 (1H, s, H-4); ^1^H-NMR (CDCl_3_, 500 MHz) of **15**: δ 0.76 (3H, s, H-18), 0.80 (3H, d, *J* = 6.8 Hz, H-27), 0.81 (3H, d, *J* = 6.8 Hz, H-26), 0.85 (3H, t, *J* = 7.2 Hz, H-29), 1.02 (3H, d, *J* = 6.8 Hz, H-21), 1.38 (3H, s, H-19), 4.35 (1H, br s, H-6), 5.03 (1H, dd, *J* = 15.2, 8.6 Hz, H-23), 5.15 (1H, dd, *J* = 15.2, 8.6 Hz, H-22), 5.82 (1H, s, H-4); ESI-MS of **14**: *m*/*z* = 451 [M + Na]^+^; ESI-MS of **15**: *m*/*z* = 449 [M + Na]^+^.

### 3.4. Biological Assay

The effect of the isolates on the neutrophil proinflammatory response was assessed by detecting the inhibition of elastase release and O_2_^•−^ generation in fMLP/CB-activated neutrophils in a concentration-dependent manner.

#### 3.4.1. Mensuration of Human Neutrophils

Human neutrophils from the venous blood of adult, healthy volunteers (20–27 years old) were isolated by a standard pattern of dextran sedimentation before centrifugation in a Ficoll Hypaque gradient and hypotonic lysis of the erythrocytes [27]. The purified neutrophils had >98% viable cells, as detected by the trypan blue exclusion method [28], were resuspended in a calcium (Ca^2+^)-free HBSS buffer at pH 7.4 and were kept at 4 °C prior to use.

#### 3.4.2. Mensuration of Superoxide Anion (O_2_^•−^) Generation

The assay for the measurement of O_2_^•−^ generation was based on the superoxide dismutase (SOD)-inhibitable reduction of ferricytochrome *c* [29,30]. In short, after supplementation with 1 mM Ca^2+^ and 0.5 mg/mL ferricytochrome *c*, neutrophils (6 × 10^5^/mL) were equilibrated at 37 °C for 2 min and incubated with varied concentrations (10–0.01 μg/mL) of either DMSO (as a control) or tested compounds **1**–**15** (purity ≥ 98%) for 5 min. Cells were incubated with cytochalasin B (1 μg/mL) for 3 min before they were activated with 100 nM formyl-l-methionyl-l-leucyl-l-phenylalanine for 10 min. Changes in absorbance with the reduction of ferricytochrome *c* at 550 nm were constantly detected in a double-beam, six-cell positioner spectrophotometer with continuous stirring (Hitachi U-3010, Tokyo, Japan). Calculations were founded on differences in the reactions with and without SOD (100 U/mL) divided by the extinction coefficient for the reduction of ferricytochrome *c* (ε = 21.1/mM/10 mm).

#### 3.4.3. Mensuration of Elastase Release

The degranulation of azurophilic granules was measured by determining elastase release as reported previously [30,31]. Assays were carried out by applying MeO-Suc-Ala-Ala-Pro-Val- *p*-nitroanilide as the elastase substrate. In brief, after supplementation with MeO-Suc-Ala-Ala-Pro-Val- *p*-nitroanilide (100 μM), neutrophils (6 × 10^5^/mL) were equilibrated at 37 °C for 2 min and incubated with tested compounds for 5 min. Cells were treated with fMLP (100 nM)/CB (0.5 μg/mL), and the changes in absorbance at 405 nm were detected constantly in order to measure elastase release. The results were displayed as the percent of elastase release in the fMLP/CB-activated, drug-free control system.

#### 3.4.4. Statistical Analysis

Results are represented as mean ± SEM, and comparisons were done by applying student’s *t*-test. A probability of 0.05 or less was deemed significant. The software SigmaPlot was employed for the statistical analysis.

## 4. Conclusions

Fifteen compounds, including a new aporphine, 3-hydroxyhernandonine (**1**), and a new lignin, 4′-*O*-demethyl-7-*O*-methyldehydropodophyllotoxin (**2**), were isolated from the resinous wood of the root wood of *H. nymphaeifolia*. The structures of these isolates were elucidated according to spectroscopic data. Granule proteases (e.g., cathepsin G, elastase) and reactive oxygen species (ROS) [e.g., hydrogen peroxide, superoxide anion (O_2_^•−^)] generated by human neutrophils gave rise to the pathogenesis of inflammatory diseases. The effects of the isolated compounds on proinflammatory responses were assessed by inhibiting fMLP/CB-induced elastase release and O_2_^•−^ generation by neutrophils. The results of anti-inflammatory assays reveal that compounds **1**–**7** and **11** can obviously inhibit fMLP-induced elastase release and/or O_2_^•−^ generation. Oxohernangerine (**5**) and 3-hydroxyhernandonine (**1**) were the most effective among the isolated compounds, with IC_50_ values of 2.65 ± 0.97 and 3.93 ± 0.48 μg/mL, respectively, against fMLP-induced O_2_^•–^ generation and elastase release. Our research indicates *H. nymphaeifolia* and its isolated compounds (especially **1**–**7** and **11**) are worth further study and may be expectantly developed as candidates for the prevention or treatment of diverse inflammatory diseases.

## Figures and Tables

**Table 1:** Inhibitory effects of compounds **1**–**15** from the root wood of *H. nymphaeifolia* on superoxide radical anion generation and elastase release by human neutrophils in response to fMet-Leu-Phe/cytochalasin B ^a^.

Compounds	Superoxide anion	Elastase
	IC_50_ [µg/mL] ^b^ or (Inh %) ^c^	
3-Hydroxyhernandonine (**1**)	4.09 ± 0.44 ***	3.93 ± 0.48 ***
4′-*O*-Demethyl-7-*O*- methyldehydro-podophyllotoxin (**2**)	5.72 ± 0.42 ***	5.40 ± 0.40 ***
2-*O*-Methyl-7-oxolaetine (**3**)	7.37 ± 0.46 ***	6.82 ± 0.09 ***
Hernandonine (**4**)	4.41 ± 0.76 ***	(45.76 ± 6.92) ***
Oxohernangerine (**5**)	2.65 ± 0.97 ***	4.82 ± 0.39 ***
Oxohernagine (**6**)	2.86 ± 0.85 ***	4.87 ± 0.27 ***
7-Oxonorisocorydine (**7**)	6.62 ± 0.28 ***	6.58 ± 0.08 ***
(–)-Deoxypodophyllotoxin (**8**)	(38.95 ± 4.83) **	(33.76 ± 3.82)
Dehydropodophyllotoxin (**9**)	(43.91 ± 3.86) ***	9.53 ± 0.84 ***
(–)-Yatein (**10**)	(42.36 ± 3.41) *	(36.74 ± 3.05) **
*N*-*trans*-Feruloylmethoxytyramine (**11**)	6.26 ± 0.65 ***	7.03 ± 0.21 ***
Mixture of β-sitostenone (**12**) and stigmasta-4,22-dien-3-one (**13**)	(24.71 ± 2.67)	(29.15 ± 2.89)
Mixture of 6β-hydroxystigmast-4-en-3-one (**14**) and 6β-hydroxystigmasta-4,22-dien-3-one (**15**)	(16.74 ± 2.66) **	7.91 ± 1.20 **
Diphenyleneiodonium ^d^	0.55 ± 0.22 ***	–
Phenylmethylsulfonyl fluoride ^d^	–	34.5 ± 5.3 ***

^a^ Results are displayed as averages ± SEM (*n* = 4). ^b^ Concentration necessary for 50% inhibition (IC_50_). If IC_50_ value of tested compound was <10 μg/mL, it was presented as IC_50_ [μg/mL]. ^c^ Percentage of inhibition (Inh %) at 10 μg/mL. If IC_50_ value of tested compound was ≥10 μg/mL, it was displayed as (Inh %) at 10 μg/mL. ^d^ Diphenyleneiodonium and phenylmethylsulfonyl were employed as positive controls for superoxide anion (O_2_^•–^) production and elastase release, respectively. * *p* < 0.05 compared with the control. ** *p* < 0.01 compared with the control. *** *p* < 0.001 compared with the control.

## Supplementary Materials

Supplementary materials are available online, Figures S1–S8: MS, 1D, and 2D-NMR spectra for 3-hydroxyhernandonine (**1**), Figures S9–S16: MS, 1D, and 2D-NMR spectra for 4′-*O*-demethyl-7-*O*-methyldehydropodophyllotoxin (**2**).
