# Supplementary material for: New Anti-Inflammatory Aporphine and Lignan Derivatives from the Root Wood of Hernandia nymphaeifolia
Source: Molecules. 2018 Sep 7;23(9):2286. doi: 10.3390/molecules23092286 (PMC6225223; doi:10.3390/molecules23092286)
Supplement: Supplementary file 1 [file molecules-23-02286-s001.pdf]

Supplementary Materials

New Anti-inflammatory Aporphine and Lignan Derivatives from the Root Wood of *Hernandia nymphaeifolia*

Chuan-Yen Wei [1,†], Shih-Wei Wang [2,†], Jin-Wang Ye [3], Tsong-Long Hwang [4], Ming-Jen Cheng [5], Ping-Jyun Sung [6], Tsung-Hsien Chang [7] and Jih-Jung Chen [8,9,*]

[1] Department of General Surgery, Taitung MacKay Memorial Hospital, Taitung City 950, Taiwan; lpshop@gmail.com

[2] Department of Medicine, Mackay Medical College, New Taipei City 252, Taiwan; shihwei@mmc.edu.tw

[3] Graduate Institute of Pharmaceutical Technology, Tajen University, Pingtung 907, Taiwan; jjc8506674@gmail.com

[4] Graduate Institute of Natural Products, School of Traditional Chinese Medicine, College of Medicine, Chang Gung University, Taoyuan 333, Taiwan; E-Mail: htl@mail.cgu.edu.tw

[5] Bioresource Collection and Research Center (BCRC), Food Industry Research and Development Institute (FIRDI), Hsinchu 300, Taiwan; cmj@firdi.org.tw

[6] National Museum of Marine Biology and Aquarium, Pingtung 944, Taiwan; pjsung@nmmba.gov.tw

[7] Department of Medical Education and Research, Kaohsiung Veterans General Hospital, Kaohsiung 813, Taiwan; changth@vghks.gov.tw

[8] Faculty of Pharmacy, School of Pharmaceutical Sciences, National Yang-Ming University, Taipei 112, Taiwan

[9] Department of Medical Research, China Medical University Hospital, China Medical University, Taichung 404, Taiwan

* Correspondence: chenjj@ym.edu.tw (J.-J.C.); Tel.: +886-2-2826-7195

† Authors have contributed equally in this manuscript.

Contents

Figure S1. ESI-MS spectrum of **1**.. S3

Figure S2. HR-ESI-MS spectrum of **1**... S3

Figure S3. ^{1}H-NMR spectrum ($CDCl_3$, 500 MHz) of **1**.. S4

Figure S4. ^{13}C-NMR spectrum ($CDCl_3$, 125 MHz) of **1**.. S4

Figure S5. ^{1}H-^{1}H COSY spectrum of **1**...S5

Figure S6. NOESY spectrum of **1**.. S5

Figure S7. HMBC spectrum of **1**... S6

Figure S8. HSQC spectrum of **1**.. S6

Figure S9. ESI-MS spectrum of **2**.. S7

Figure S10. HR-ESI-MS spectrum of **2**.. S7

Figure S11. ^{1}H-NMR spectrum ($CDCl_3$, 500 MHz) of **2**... S8

Figure S12. ^{13}C-NMR spectrum ($CDCl_3$, 125 MHz) of **2**.. S8

Figure S13. ^{1}H-^{1}H COSY spectrum of **2**. S9

Figure S14. NOESY spectrum of **2**.. .... S9

Figure S15. HMBC spectrum of **2** S10

Figure S16. HSQC spectrum of **2**.. S10

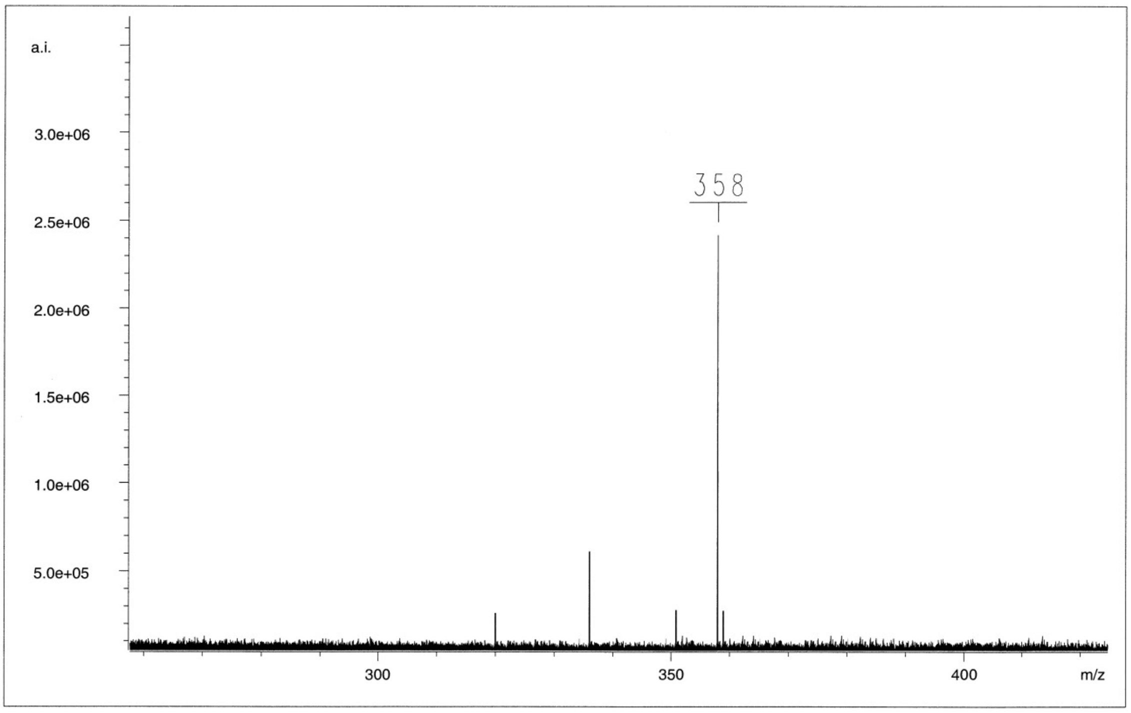

Figure S1. ESI-MS spectrum of **1**.

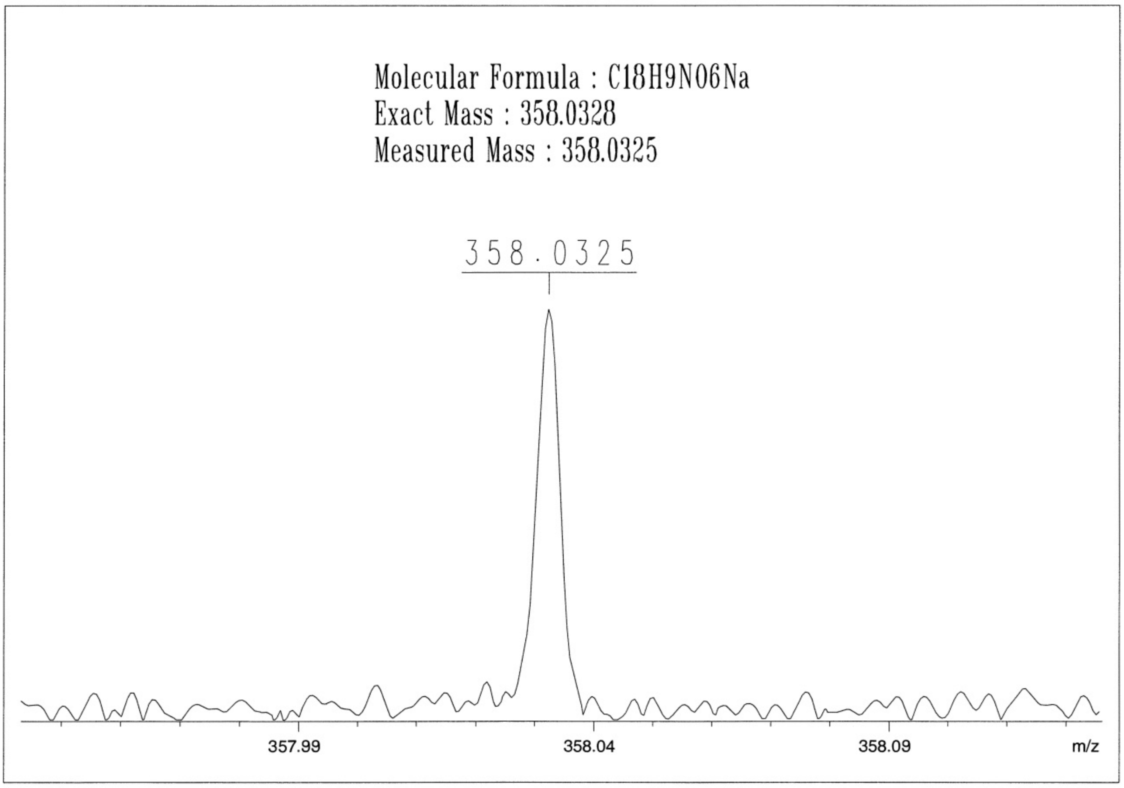

Figure S2. HR-ESI-MS spectrum of **1**.

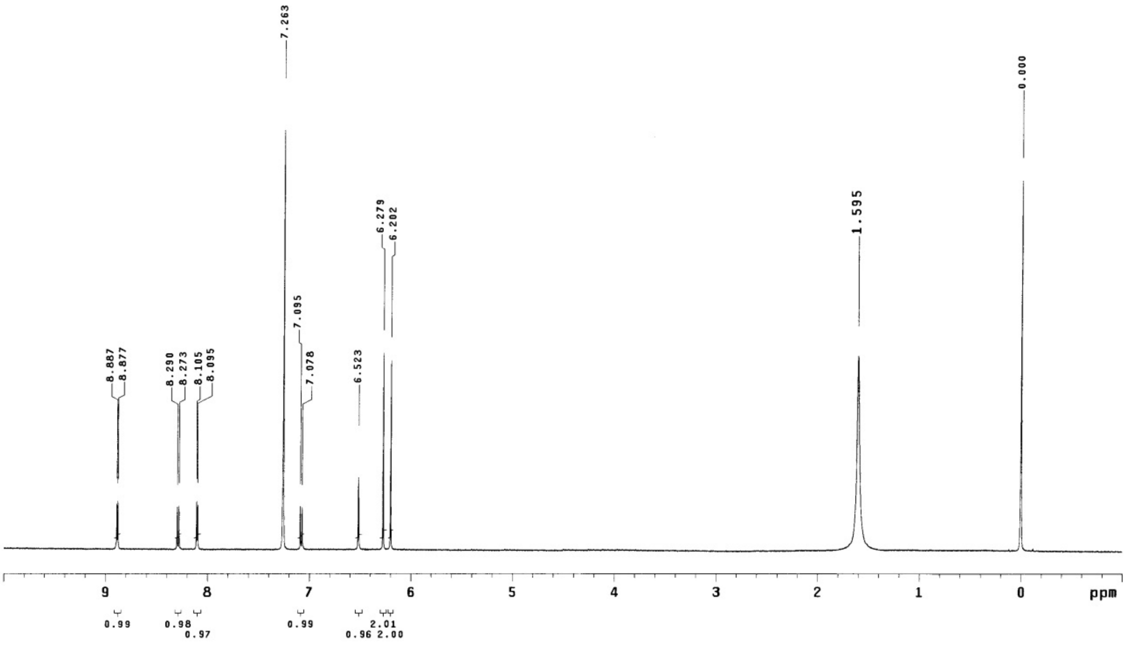

Figure S3. ^{1}H-NMR spectrum ($CDCl_3$, 500 MHz) of **1**.

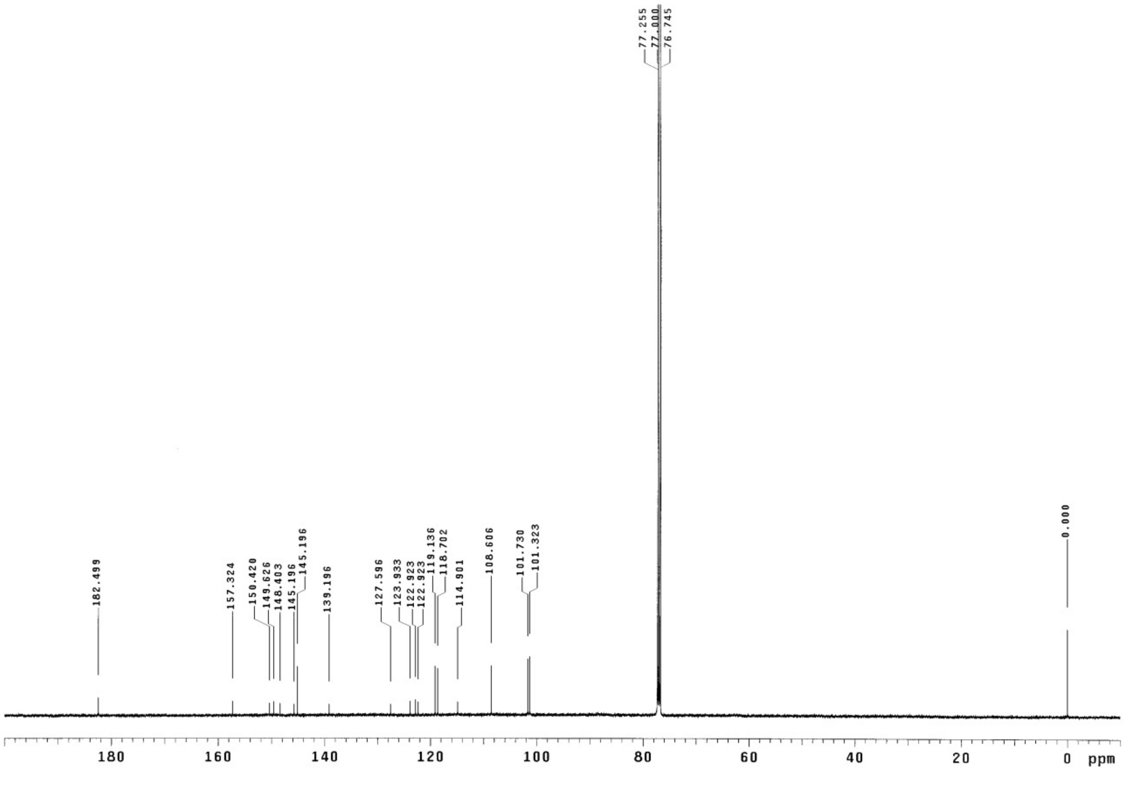

Figure S4. ^{13}C-NMR spectrum ($CDCl_3$, 125 MHz) of **1**.

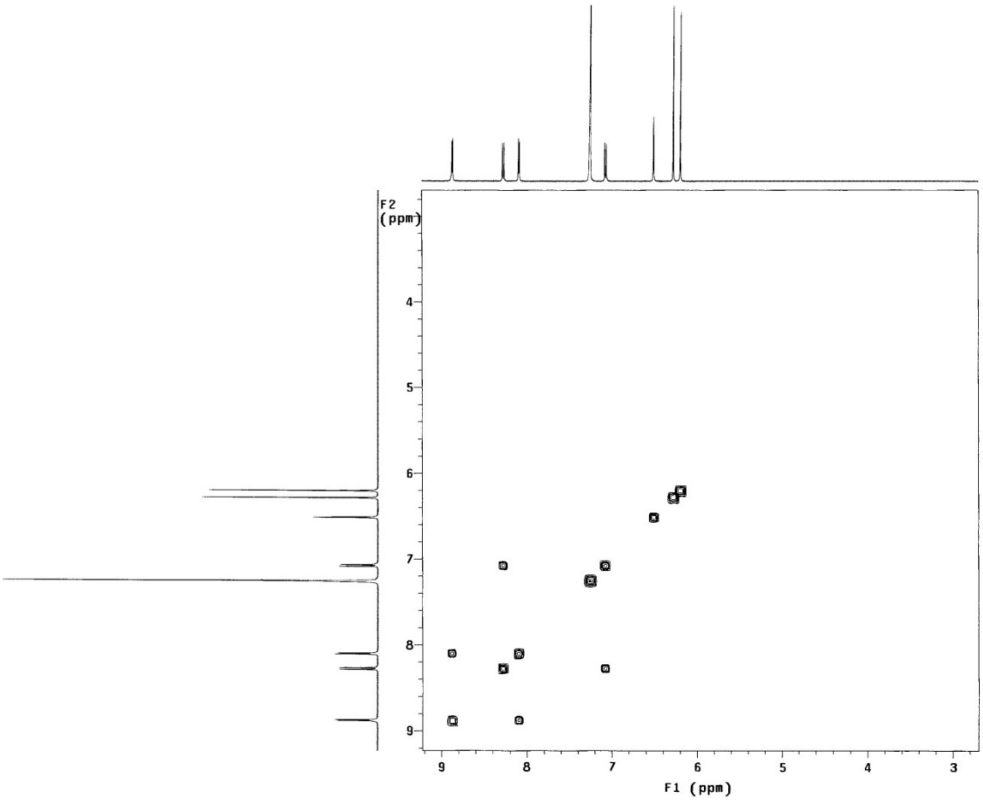

Figure S5. ^{1}H–^{1}H COSY spectrum of **1**.

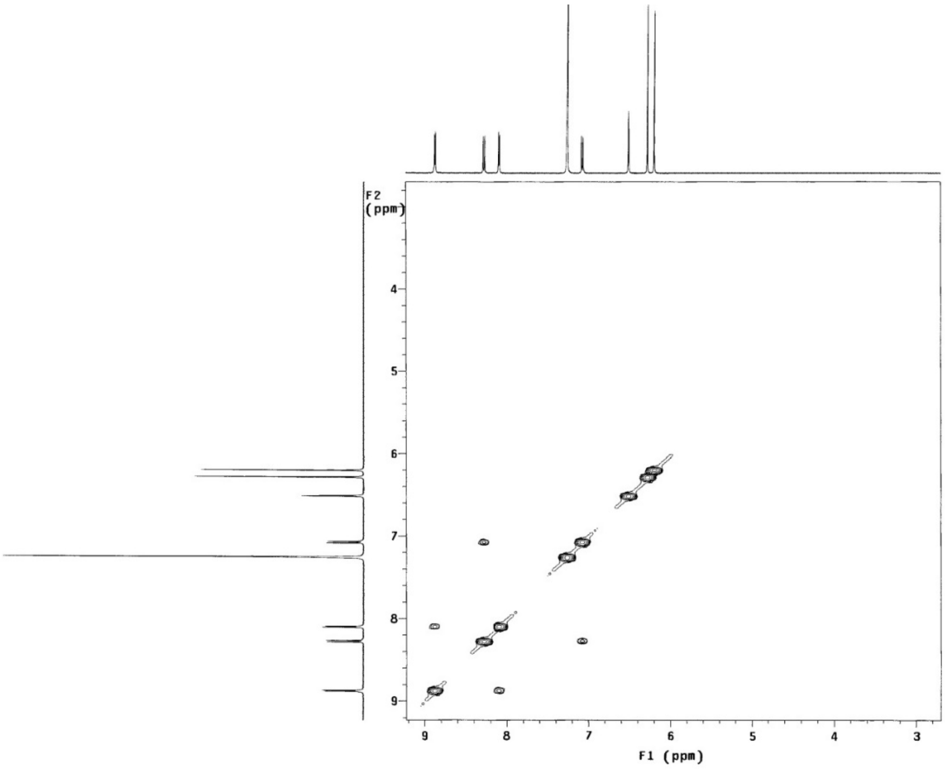

Figure S6. NOESY spectrum of **1.**

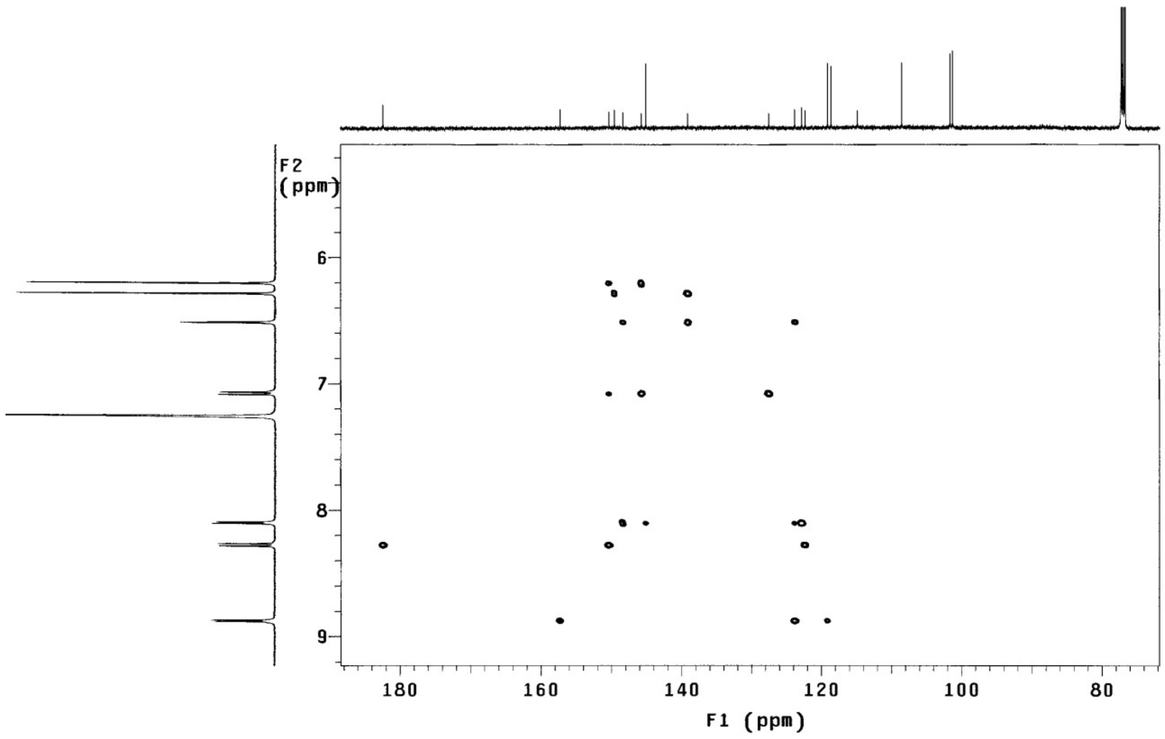

Figure S7. HMBC spectrum of **1**.

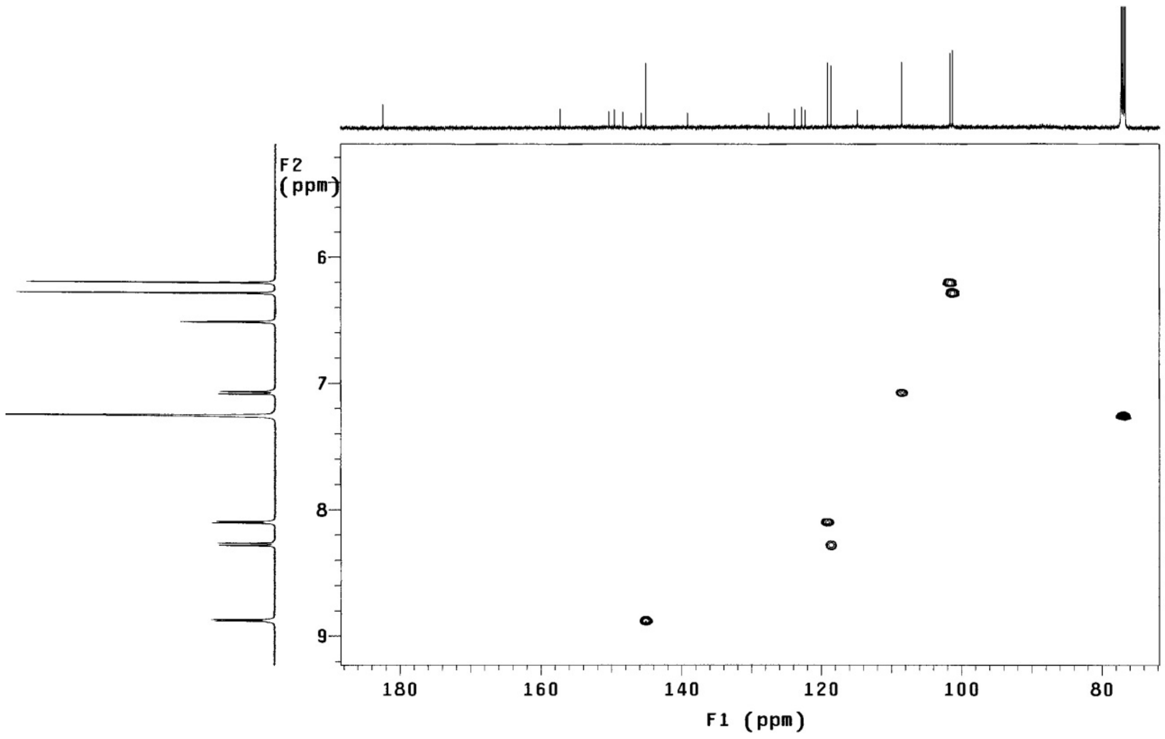

Figure S8. HSQC spectrum of **1**.

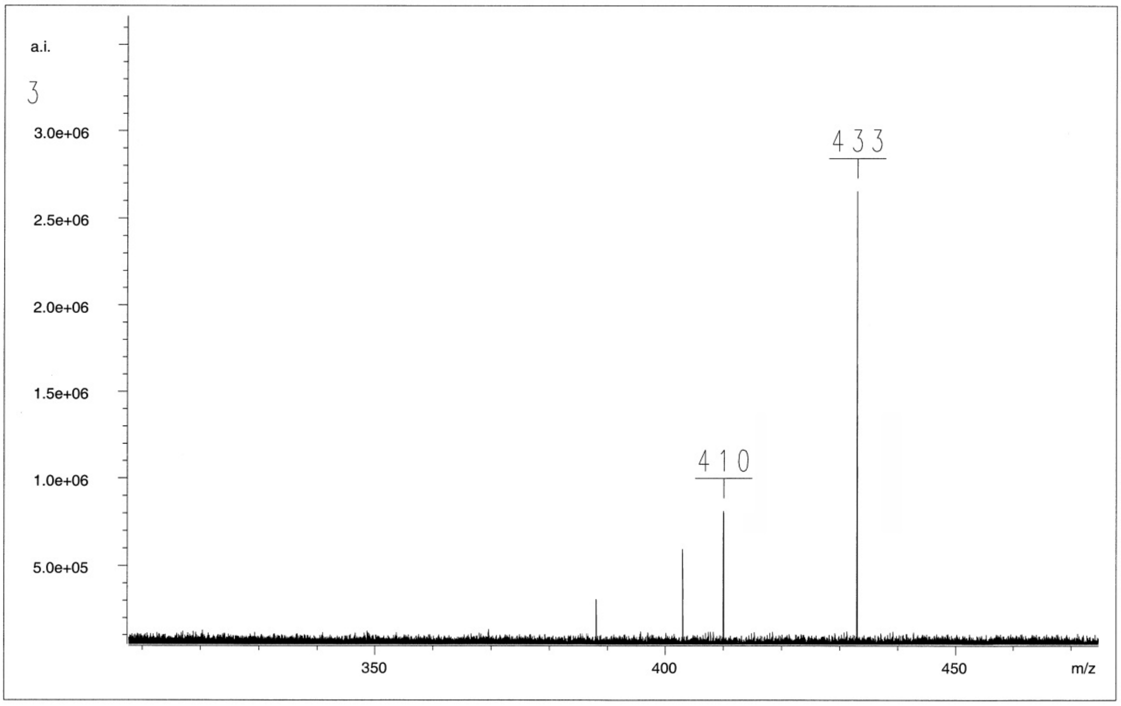

Figure S9. ESI-MS spectrum of **2**.

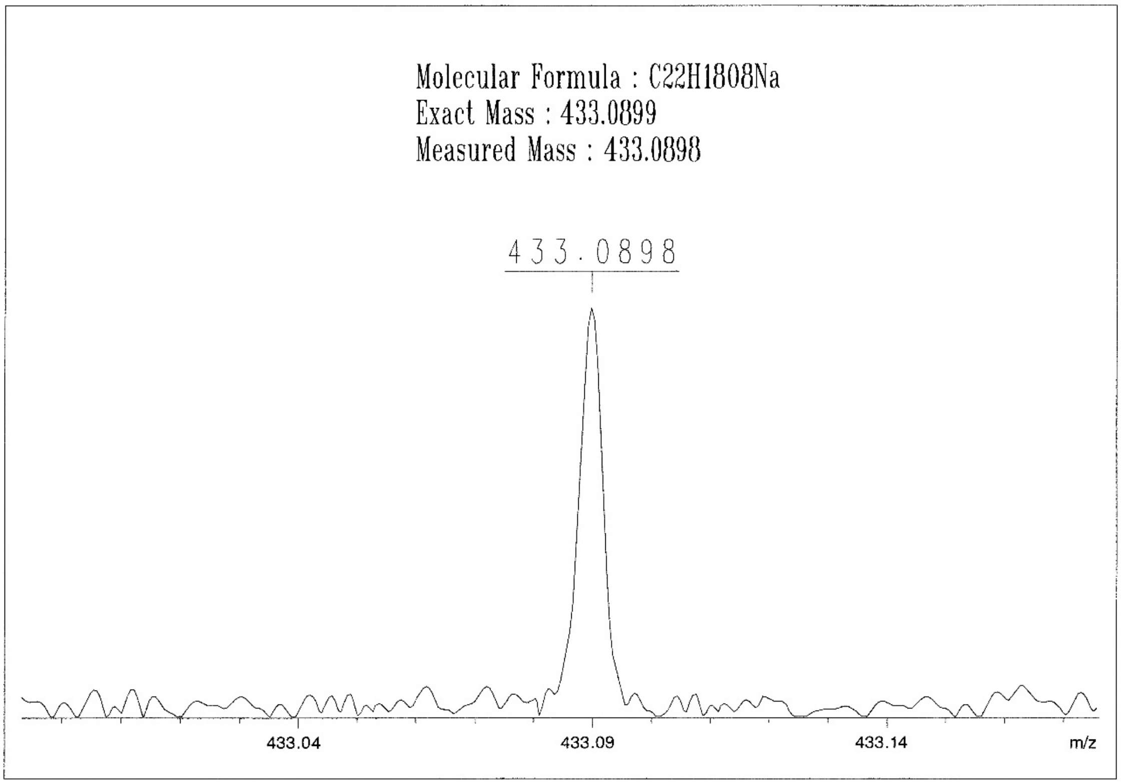

Figure S10. HR-ESI-MS spectrum of **2**.

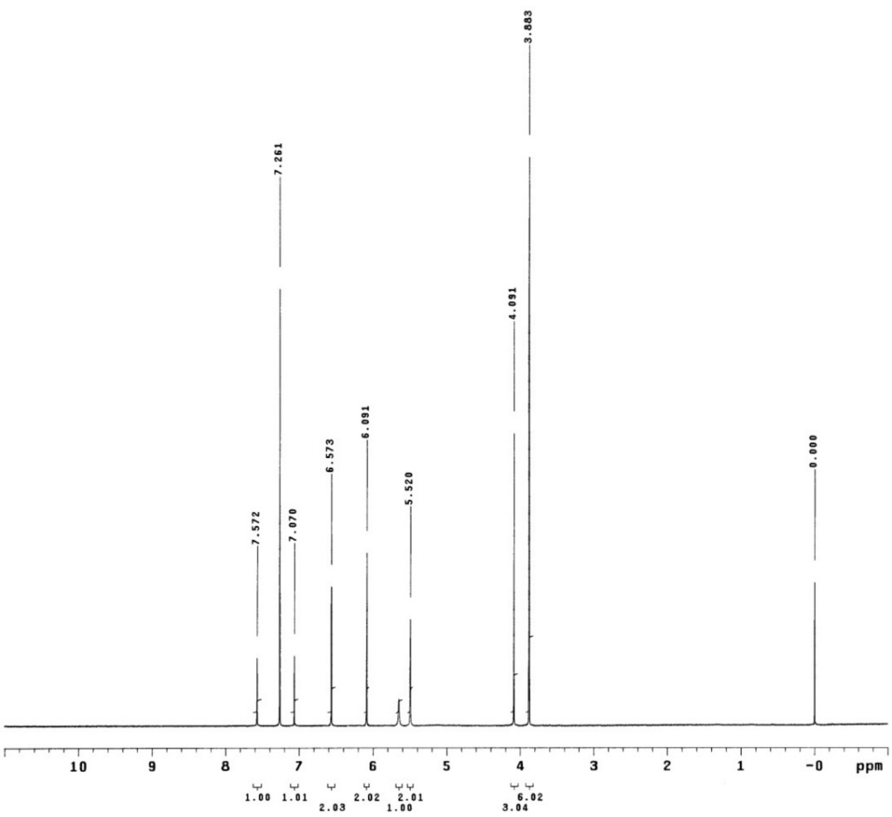

Figure S11. ^{1}H-NMR spectrum ($CDCl_3$, 500 MHz) of **2**.

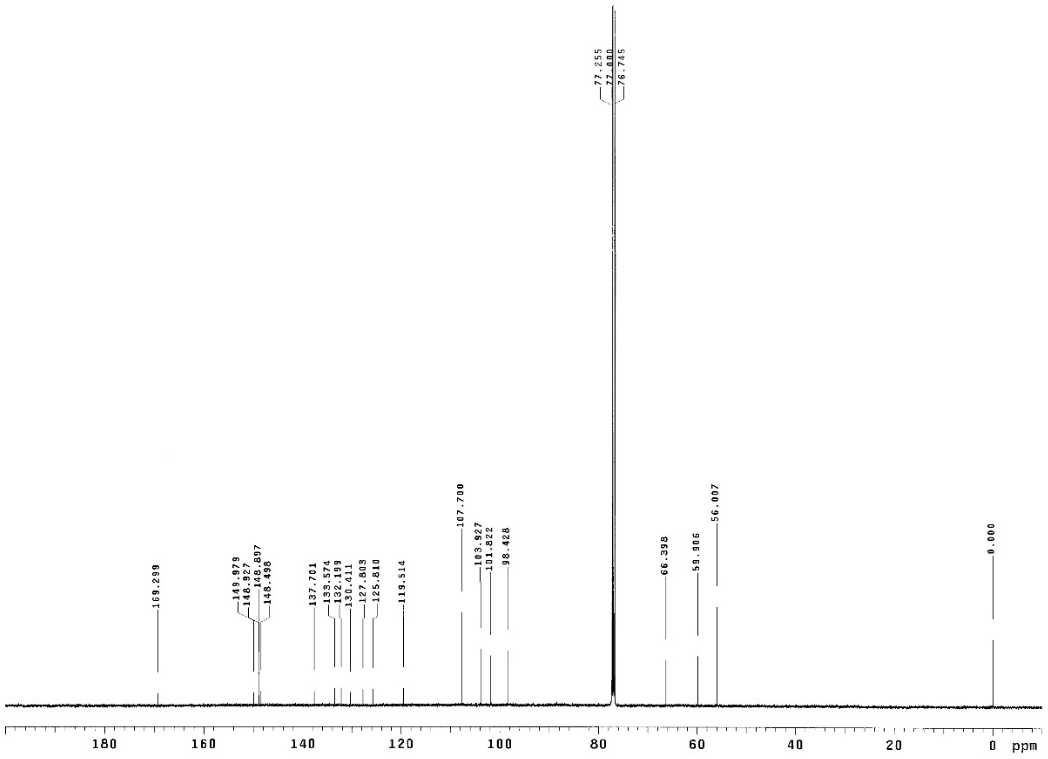

Figure S12. ^{13}C-NMR spectrum ($CDCl_3$, 125 MHz) of **2**.

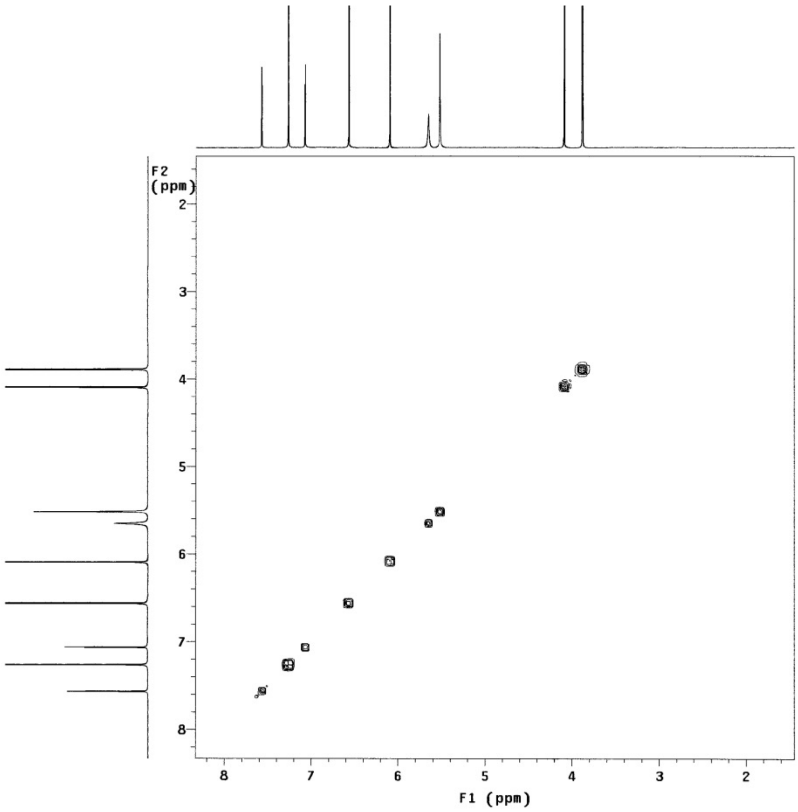

Figure S13. ^{1}H–^{1}H COSY spectrum of **2**.

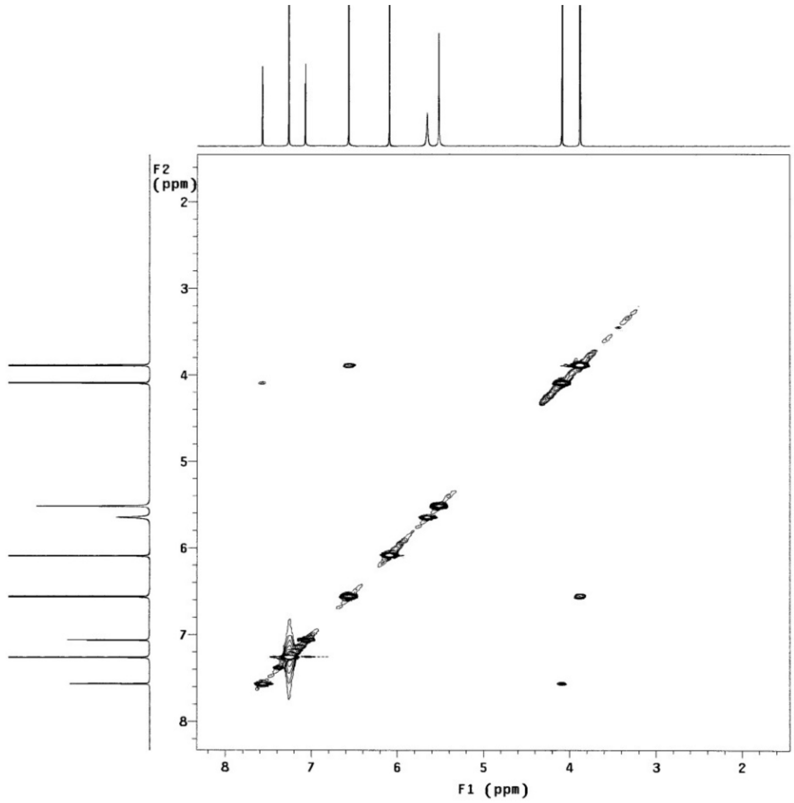

Figure S14. NOESY spectrum of **2**.

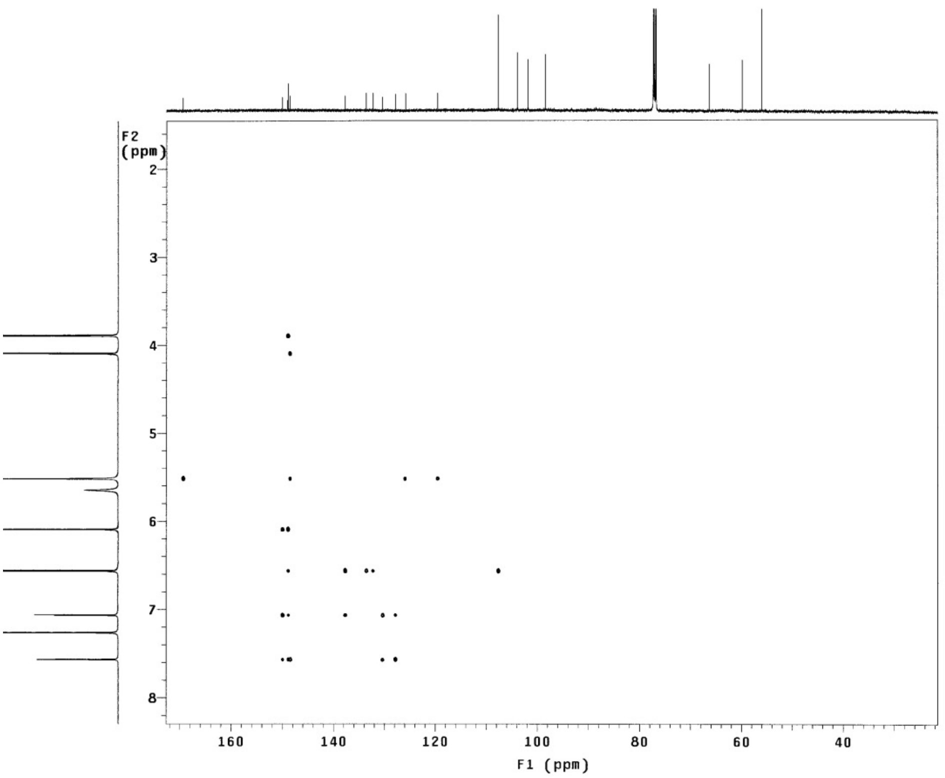

Figure S15. HMBC spectrum of **2**.

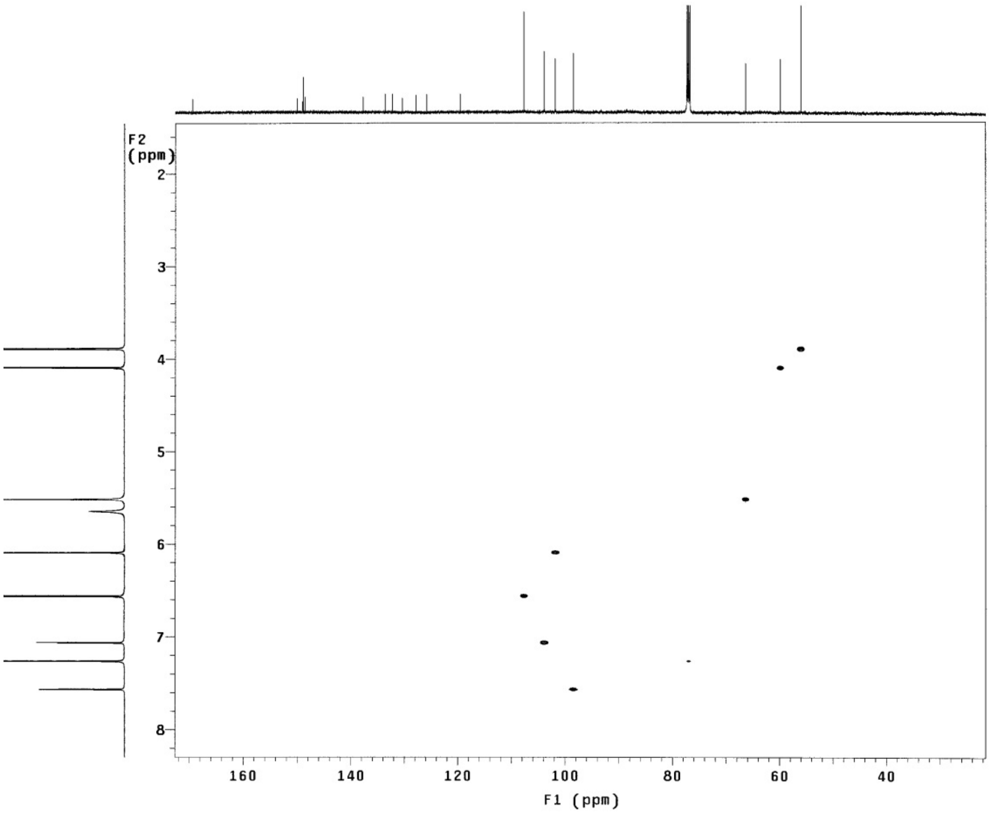

Figure S16. HSQC spectrum of **2**.
